# TCM-Mesh: The database and analytical system for network pharmacology analysis for TCM preparations

**DOI:** 10.1038/s41598-017-03039-7

**Published:** 2017-06-06

**Authors:** Run-zhi Zhang, Shao-jun Yu, Hong Bai, Kang Ning

**Affiliations:** Key Laboratory of Molecular Biophysics of the Ministry of Education, Hubei Key Laboratory of Bioinformatics and Molecular-imaging, Department of Bioinformatics and Systems Biology, College of Life Science and Technology, Huazhong University of Science and Technology, Wuhan, Hubei 430074 China

## Abstract

With the advancement of systems biology research, we have already seen great progress in pharmacology studies, especially in network pharmacology. Network pharmacology has been proven to be effective for establishing the “compounds-proteins/genes-diseases” network, and revealing the regulation principles of small molecules in a high-throughput manner, thus would be very effective for the analysis of drug combinations, especially for TCM preparations. In this work, we have proposed the TCM-Mesh system, which records TCM-related information collected from various resources and could serve for network pharmacology analysis for TCM preparations in a high-throughput manner (http://mesh.tcm.microbioinformatics.org/). Currently, the database contains 6,235 herbs, 383,840 compounds, 14,298 genes, 6,204 diseases, 144,723 gene-disease associations, 3,440,231 pairs of gene interactions, 163,221 side effect records and 71 toxic records, and web-based software construct a network between herbs and treated diseases, which will help to understand the underlying mechanisms for TCM preparations at molecular levels. We have used 1,293 FDA-approved drugs, as well as compounds from an herbal material *Panax ginseng* and a patented drug Liuwei Dihuang Wan (LDW) for evaluating our database. By comparison of different databases, as well as checking against literature, we have demonstrated the completeness, effectiveness, and accuracy of our database.

## Introduction

Traditional Chinese Medicine (TCM) has been widely used in clinics in China for at least 3,000 years. Through continuous development and innovation, ancient physicians select the essence and discard the dross of TCM, the TCM theory forms a unique academic system finally. The traditional Chinese system of medicine is a complex system, which not only contains medicine plants but also includes animal and mineral materials. With their respective dosages by the guidance of Chinese medicine theory and the rule of “King, Vassal, Assistant and Delivery servant”^1^, the TCM preparation (or patented drug) was produced. And particular attention should be focused on the materials and the progress of preparation to ensure its efficacy and safety.

Nowadays, many traditional Chinese herbs are famous for their therapeutic effect and low toxicity around the world and have increasingly arisen people’s attention^2–4^. For example, *Ganoderma lucidum*, called ‘Ling-Zhi’ in China, has been used as a health-preserving and therapeutic agent. Pharmacological studies have shown that *G. lucidum* possesses medicinal properties including anti-cancer, anti-diabetic, anti-hepatotoxic and immunomodulatory^5–8^. The polysaccharides of *G. lucidum*, as the major bioactive ingredients, are responsible for the therapeutic uses. Additionally, *Panax ginseng* is a traditional herbal medicine that has been used therapeutically for more than 2,000 years. As the most valuable of all medicinal plants, especially in China, Korea, and Japan, ginseng exert a wide range of medicinal efficacy. Currently, it has been used to treat various diseases such as cardiovascular diseases^9, 10^, Alzheimer disease^11^, sleep disorder and diabetes^12, 13^. Ginseng contains a considerable amount of different compounds, of which the ginsenoside plays a major role^14^. In addition to single traditional herbs, Liuwei Dihuang Wan (LDW), a representative famous Chinese medicine preparation, is consist of six herbs^15, 16^: *Rehmannia glutinosa* libosch., *Cornus officinalis* Sieb., *Dioscorea oppositifolia* L., *Paoenia ostii*, *Alisma orientale* Juz and *Poria cocos* (Schw.) *Wolf*, with a dose proportion of 8:4:4:3:3:3^17, 18^. So far, LDW has remained one of the most popular TCM preparations prescribed for primary or adjuvant treatment of many kinds of diseases, such as diabetes, hypertension, diabetic retinopathy and nephropathy^19–24^.

The usage for TCM preparations has been reasonable and extensive in recent years, but many problems remain to be resolved, such as quality control and therapeutic targets evaluation. Unlike modern drugs discovered by targeting a specific protein, understandings of the molecular basis of TCM are still limited, it is a serious challenge for the modernization of TCM^3^. As analytical theory and technology advanced rapidly, the TCM studies must keep up the pace of modern science to remain powerful. Since the effects on human cells and genes by the chemical ingredients from TCM remain unclearly, deep mining of related information is necessary. There is no doubt that the investigation of the molecular basis of herbal formulae will increase the acceptance of TCM worldwide^25^.

With the development of the big data research in biomedicine, network pharmacology has emerged as a systematic approach for drug-target analysis, and it has gradually pushed the paradigm shift for drug discovery: the shift from “one target, one drug” mode to “network target, multiple component therapeutics” mode^26–28^. The network pharmacology approach has been abstracted as the interpretation of the “compound-proteins/genes-diseases” pathways (chains). And it is capable of describing complex interactions among biological systems of the human body, drugs, and diseases from a network perspective. For instance, if a compound and an approved drug target the same protein, the compound may be the most promising candidate for the discovery of the new drug, and if a compound and a disease share the same protein, the TCM that contains the compound as main constituent may have the same effect on this disease. Since the holistic philosophy of TCM shares the key ideas with network pharmacology, a new interdisciplinary termed TCM network pharmacology has been proposed^28–30^, which provides a new research paradigm for translating TCM from an experience-based medicine to an evidence-based medicine system.

For the treatment of disease, TCM functions as a whole. That is, each herbal formula has its corresponding syndromes. While in the modern science, the synergy between diverse compounds of a medicine is the basis of its function. Network pharmacology studies can help people understand the role of a herbal formula in molecular level. Moreover, by analyzing the “drug-target-disease” interaction network in medication process, the researchers can reveal the effects of the herbal formula systematically. In summary, the TCM network pharmacology proposed has not only become an appropriate entry point for modern TCM research but also provided a strong theoretical support for the discovery of new indications of TCM.

Currently, various databases have been used for TCM analysis: Super Natural II^31^, TCM@taiwan^32^, ChEMBL^33^ and TCMID^34^ are mainly for compounds of TCMs; STITCH^35^ is for compound-protein interactions; STRING^36^ is for protein-protein interactions; OMIM^37^ and GAD^38^ are for protein-disease associations. However, network pharmacology analysis is limited by several aspects of currently available databases. For example, database such as TCMID is not complete enough, whose compounds are much less than STITCH. In addition, a large amount of redundancies from databases including OMIM and STITCH have to be trimmed to improve the efficiency of the database. Finally and most importantly, the independent database can’t fully reflect the medicinal compounds’ effects on human. Therefore, more comprehensive and optimized database and analytical system for TCM are needed, which will help us better understand the therapeutic mechanism of the TCM preparation. And for the comprehensive understanding of the effects of TCMs, toxicity^39^ and side effects^40, 41^ databases are crucial for the safety assessment: For many herbs, it’s their toxicity that is used to treat diseases; for other herbs, although they are non-toxicity or low toxicity, overdosing will lead to serious side reaction. For example, it was reported that 121 people died in the past 60 years due to the overdose of Ephedra^42^. Therefore, it’s of great importance for deciphering the toxicity mechanism of TCMs.

In this work, we have proposed the TCM-Mesh platform, which was designed as an integration of database and a data-mining system for network pharmacology analysis of TCM preparations. The TCM-Mesh system has realized the network pharmacology analysis in a comprehensive, one-stop manner. Thus it could well serve for the network pharmacology analysis, especially for TCM preparations. TCM-Mesh recorded about > 6,000 herbs, >380,000 compounds, >14,000 genes, >6,000 diseases, >140,000 gene-disease associations, >160,000 side effect records and >70 compound toxic records. Through the online interface offered by the TCM-Mesh web portal, users could make an inquiry that facilitated the information mining of TCMs from multiple perspectives, as well as downloading those information for further analysis locally.

## Results and Discussions

### General statistics of TCM-Mesh

Through data integrating and filtering, TCM-Mesh now contained up to 6,235 herbs, 383,840 compounds, 4,518,065 compound-gene interactions, 14,298 genes, 3,440,231 gene interactions, 6,204 diseases, 144,723 gene-disease associations, 163,221 side effect records and 71 toxicity records. The combined score (0–999), obtained from the STITCH, represented the strength of the links between the compounds and the different proteins. The computing method was recorded in the literature^43, 44^, and proteins/genes with combined score higher than 700 were regarded as compound’s predicted functional partners. Notice that TCM-Mesh was mainly developed for finding the “compounds-proteins/genes-diseases” pathways, the herb-related compounds without Can SMILE string and CID number were removed from our database, and as a result, the number of herbs was reduced inevitably.

We compared TCM-Mesh with TCMID and STITCH databases. Now TCMID records about 46,929 prescriptions, 8,203 herbs, 43,413 compounds and 3,854 diseases according to the home page of TCMID. We have found that the data size in TCM-Mesh were much more than in the TCMID (Table 1). This is due to the fact that as we recorded not only the basic herb information, the compound-proteins/genes-diseases pathway of the ingredients but also the toxicity records, the side effects records, as well as the gene interactions that were not included in TCMID. Compared to STITCH, while STITCH contained up to 45,124,160 compounds, TCM-Mesh only contained 383,840 compounds, which was significantly smaller.

**Table 1:** Comparisons of data sizes between TCM-Mesh and TCMID. Comparisons were made from four aspects: herbs, compounds, diseases and compound-protein interactions by comparing the size between TCM-Mesh and TCMID.

Data fields	The amount of data in TCM-Mesh	**The amount of data in TCMID**
Herbs	6,235	8,203
Compounds	383,840	43,413
Compound-protein interactions	4,518,065	211,152
Disease	6,204	3,854

### Assessment of database

#### Evaluation accuracies of database

Eight drugs used for the treatment of intestinal diseases: Eight Intestinal diseases related drug were selected manually to test TCM-Mesh. They were Leucovorin, Oxaliplatin, Regorafenib, 5-Fluorouracil, Capecitabine, Sulfasalazine, Prednisone and Hydrocortisone respectively (Table 2).

**Table 2:** Eight drugs used for intestinal diseases. The drug names of 5-Fluorouracil and Hydrocortisone could not be searched in database, the synonyms of these drugs were provided.

Drug name	Synonym	Related diseases	**Candidate related genes**
Hydrocortisone	Cortisol	Inflammatory disease	CRH, CYP19A1, HSD11B1, POMC, SERPINA6
5-Fluorouracil	5-FU	Colorectal cancer and breast cancer	ABCC5, CASP3, CASP8, CYP2C9, DHFR, DPYD, TP53, TYMP, TYMS
Leucovorin	/	Colorectal cancer	ABCC2, MTHFS, RFC1, SHMT1
Regorafenib	/	Gastrointestinal stromal tumors	BRAF, FLT1, KDR, KIT, PDGFRA
Capecitabine	/	Colorectal cancer and breast cancer	CDA, CYP2C9, CYR61, DPYD, HIF1A, HRAS, TYMS
Sulfasalazine	/	Inflammatory bowel disease	ABCG2, IL1B, MPO, NAT2, NFKBIA, PTGS2
Prednisone	/	Inflammatory disease, some autoimmune diseases and some types of cancer	ALB, CRP, CYP3A4, HLA-A, HLA-C, NR3C1
Oxaliplatin	/	Colon cancer	BIRC5, CASP3, CCND1, ERCC1, GSTP1, PARP1, VEGFA

We used the drug name to search with the “compounds-proteins/genes-diseases” network been retrieved from the TCM-Mesh. For 5-fluorouracil and Hydrocortisone, they were not available in our database, as we used their respective synonyms: 5-FU and Cortisol for the alternatives. For each drug, we retained the 10 genes with the highest combined score for simplicity. And we aimed to figure out whether the diseases extracted from our database matched with the indication of the drug, and furthermore, which gene was the candidate target for the drug. The detailed information of candidate genes of drugs was shown in Table 2. Leucovorin was used for treat advanced colorectal cancer^45, 46^. According to the TCM-Mesh, the Leucovorin related-gene were ABCC2, ABCC4, AMT, FTCD, MTHFD2, MTHFD2L, MTHFS, RFC1, SHMT1 and SHMT2 respectively, four of them were related to colorectal cancer (the gene-disease associations were obtained from the TCM-Mesh, the same below). For 5-fluorouracil, it is effective on colorectal cancer^46^ and breast cancer^47^. And its related genes were ABCC5, CASP3, CASP8, CYP2C9, DHFR, DPYD, TP53, TYMP, TYMS and UPP1 respectively according to the TCM-Mesh, all genes were related to colorectal cancer except UPP1. Hydrocortisone was used to treat various kinds of inflammatory disease such as arthritis^48, 49^. Its related genes were CRH, CYP19A1, CYP3A4, HSD11B1, HSD11B2, HSD3B2, NR3C1, POMC, SERPINA6 and SHBG, of which CRH, CYP19A1 and SERPINA6 were related to arthritis, as well as HSD11B1 and POMC were related to inflammation. Regorafenib was reported to cure gastrointestinal stromal tumors^50, 51^, its related genes were BRAF, CSF1R, FLT1, KDR, KIT, PDGFRA, RAF1 and RET respectively. According to the TCM-Mesh, more than half of them were related to colorectal cancer and gastrointestinal stromal tumors. Capecitabine was used to cure colorectal cancer and breast cancer^47, 52^, and its related genes were CDA, CES1, CYP2C9, CYR61, DPYD, ERBB3, HIF1A, HRAS TYMP and TYMS respectively, seven of which were related to colorectal cancer and breast cancer. Sulfasalazine was used for the treatment of inflammatory bowel disease^53^, whose related genes were ABCG2, ALOX5, CRP, IL1B, MPO, NAT2, NFKBIA, PTGS2, SLC46A1 and SLC7A11 respectively. And six of them were related to the indication of Sulfasalazine. Prednisone was a synthetic corticosteroid drug that was used to treat certain inflammatory disease (such as moderate allergic reactions), as well as some autoimmune diseases and several types of cancers (at higher doses)^54, 55^. Its related genes were ABCB1, ALB, CRP, CYP3A4, HLA-A, HLA-C, HSD11B1, INS, NR3C1 and SERPINA6 respectively. And out of these genes, NR3C1 was related to atopic asthma, CRP was associated with inflammation, ALB was a C-reactive protein which was regarded as a marker of inflammation, HLA-A and HLA-C were related to autoimmune disease, as well as CYP3A4 was related to prostate cancer. Oxaliplatin, whose ten related genes were BIRC5, CASP3, CCND1, ERCC1, GSTP1, MTOR, PARP1, SRC, TYMP and VEGFA respectively, was used for curing colon cancer^45, 52^. And seven of these genes were related to the indication.

**Figure 1:**
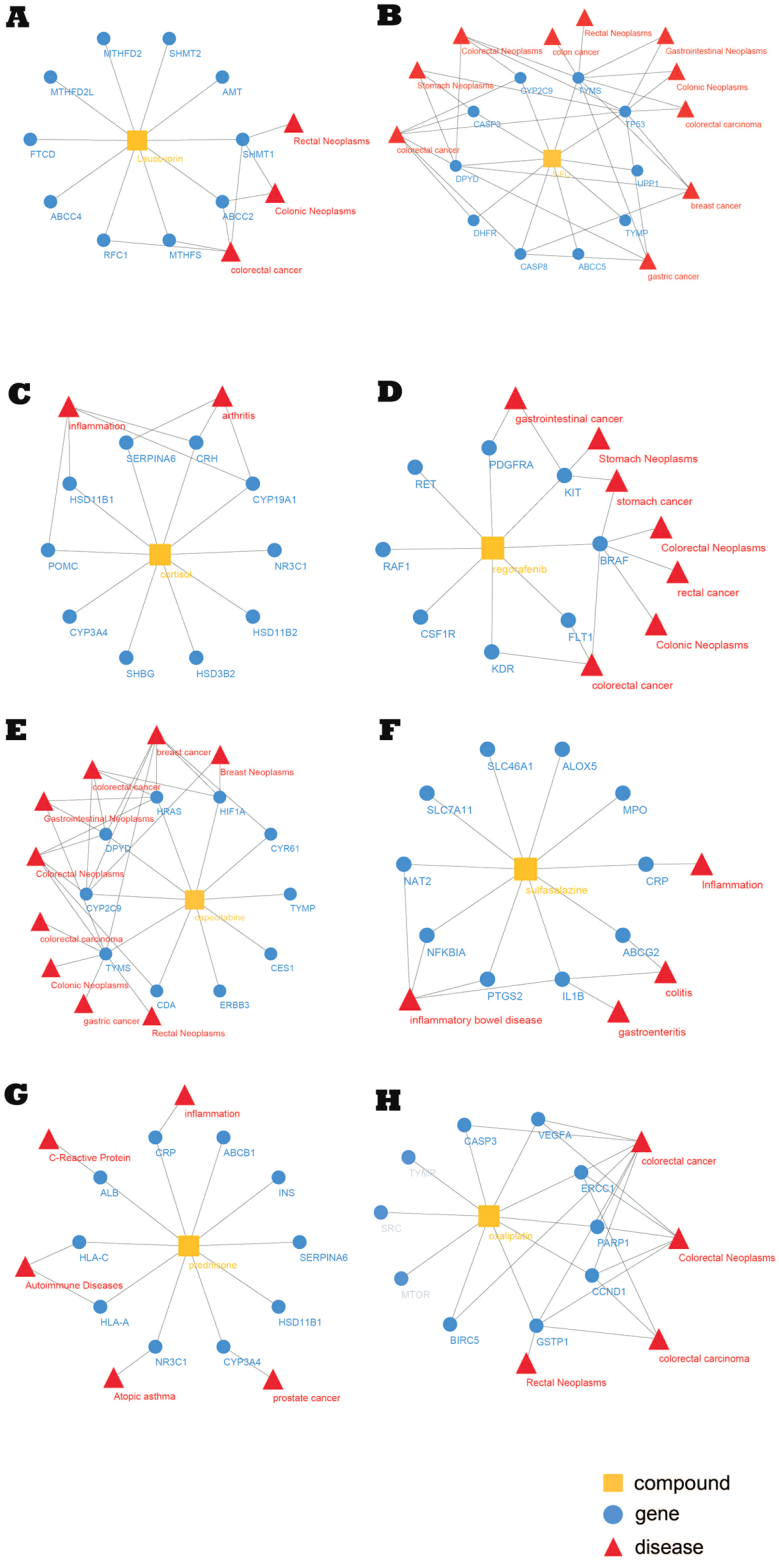
The network constructed for eight FDA approved drugs. (**A**) Leucovorin; (**B**) 5-fluorouracil; (**C**) Hydrocortisone; (**D**) Regorafenib; (**E**) Capecitabine; (**F**) Sulfasalazine; (**G**) Prednisone; (**H**) Oxaliplatin. The innermost node represented the drug, the circle in the middle represented the drug-related genes, the outermost layer represented the gene related diseases. Node: yellow square, compound; blue circle, gene; red triangle, disease. Line: the line between yellow square and blue circle, compound-gene links; the line between blue circle and red triangle, gene-disease associations.

As shown in Fig. 1, for all of the eight drugs, each of which had at least 4 related genes whose candidate related-disease matched with the indications of the drugs.

#### FDA approved drugs

**Figure 2:**
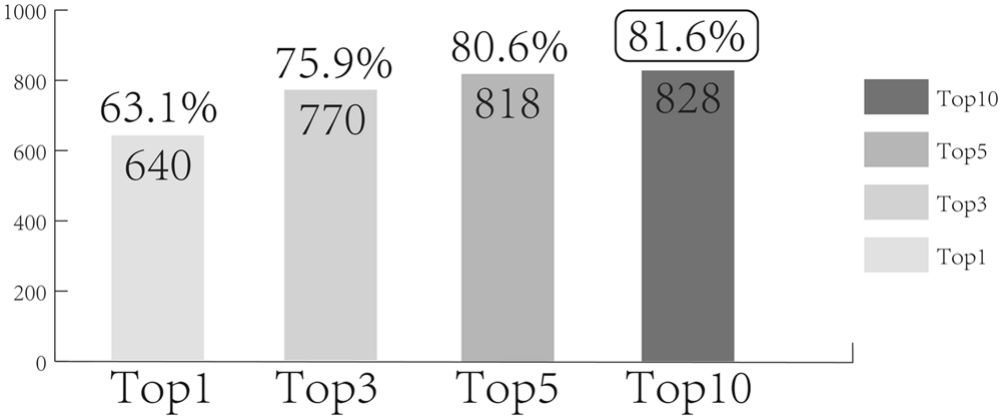
Evaluation of database accuracy based on search results. Different histograms represented top-n genes with the highest combined score of a specific drug used for evaluation; the number in the histogram represented the number of drugs whose top-n genes were related to the indication; percentage represented the ratio of the number in the histogram to the amount of drugs used for evaluation. X axis: top-n genes that were used for evaluation; Y axis: number of the drugs whose related genes were related to the indication of the drug.

To further evaluate the effectiveness of the database, 1,293 FDA approved small molecular drugs were retrieved from the DrugBank^56^. Firstly, 1,293 compounds were used to search in the TCM-Mesh with 1,108 compounds and 103,428 related proteins been retrieved from the database. Secondly, as too many results were listed when we set combined score to 700, we set combined score to 800 for simplicity. We used proteins for searching with 16,061 approved genes (1,015 compounds were retained) been collected. For each of drugs, we have chosen 10 genes with the highest score. By counting manually, we found that for these 1,015 FDA approved small molecular drugs, 63.1% (640) of whose highest scoring gene was associated with the indications of the drug (the gene-disease associations were obtained from the TCM-Mesh, the same below), and 75.9%, 80.6%, 81.6% of whose top3, top5, top10 highest scoring genes were related to the indications of the drug respectively (Fig. 2).

From the Fig. 2, potential “compound-proteins/genes-diseases” pathways of 828 drugs (81.6% of 1,015 drugs) were retrieved from the database. Nowadays, cardiovascular disease has been regarded as one of the most common diseases for human health. Modern scientists have spent a lot of time studying cardiovascular disease and finding its related therapeutic targets. In our test, 16% of the drugs (162 out of 1,015) were used for curing cardiovascular diseases with potential targets of 96% drugs been retrieved from the TCM-Mesh.

#### Completeness of database

**Figure 3:**
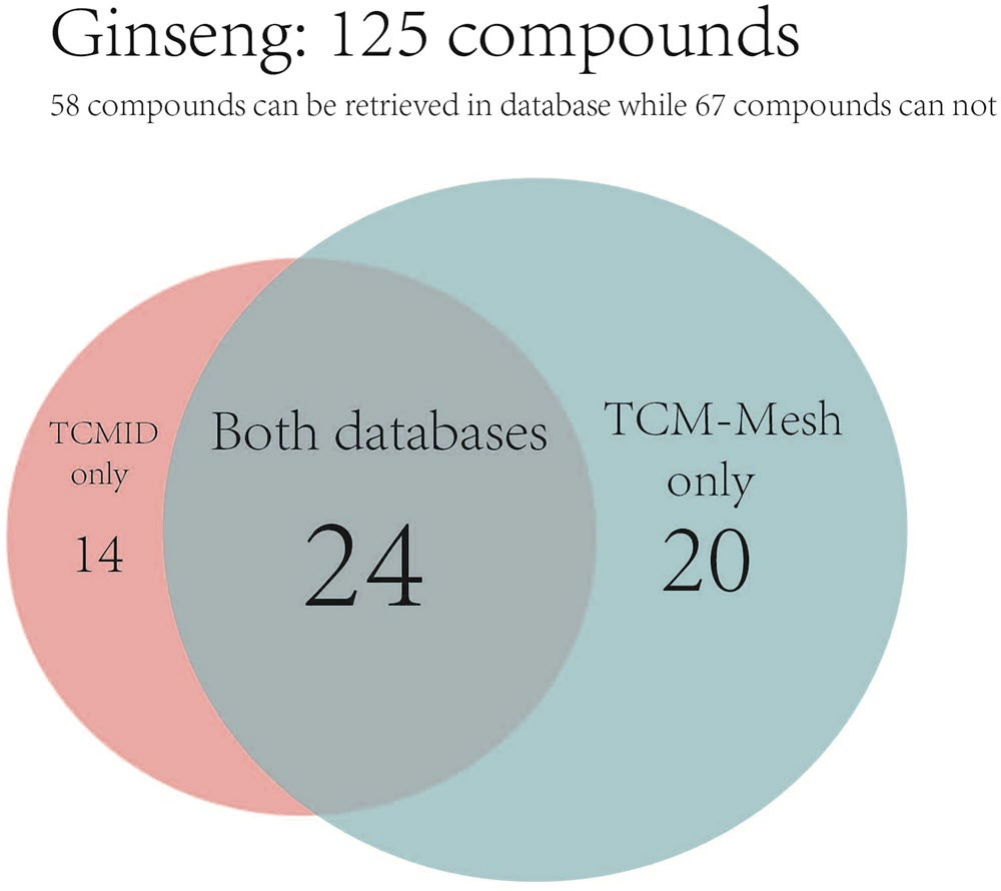
The comparison result of TCM-Mesh and TCMID by using compounds of ginseng. The number in the circle represented the amount of compounds that could be retrieved with hits in the databases.

Comparison with TCMID: To evaluate the completeness of TCM-Mesh, we compared our database with TCMID. We used the compounds of ginseng for testing in both databases. 125 compounds of ginseng were collected from TCM@Taiwan database. We used each compound for searching in both databases, the results showed that 38 out of 125 compounds could be searched with hits from TCMID, and 44 out of 125 compounds could be searched with hits in TCM-Mesh (Fig. 3). This has shown the advantage of TCH-Mesh as it is a more complete database compared to TCMID.

#### Comparison with STITCH

In addition to TCMID, we also compared our database with one of our source databases STITCH. The data of compound-proteins were obtained from the STITCH database. We used the 1,293 FDA-approved drugs and 125 compounds of ginseng mentioned above for testing (Table 3), with results showing that 1,108 out of 1,293 compounds could be searched with hits from STITCH. Furthermore, the 44 out of 125 compounds of ginseng could be searched with hits from STITCH. Interestingly, the TCM-Mesh shared exactly the same searching results with STITCH while the data size of TCM-Mesh was much smaller.

**Table 3:** Comparison of searching results between TCM-Mesh and STITCH. 1,293 FDA approved drugs and compounds of ginseng were searched in both databases and retrieved corresponding proteins, the searching results were shown in the table, and the number of compounds that could be searched in both databases was shown as identical. The size of both databases was for auxiliary comparison.

Completeness test	TCM-Mesh	STITCH-Protein	Identical
1293 FDA approved drugs	1,108/1,293	1,108/1,293	1,108
Compounds of ginseng	44/125	44/125	44
Amount of compounds	383,840	45,124,160	/

### Assessment of networks

#### Assessment of networks generated for ginseng

We used the name and aliases of 125 compounds from ginseng for searching, and as a result, we retrieved 5,183 pairs of associations between 44 compounds and 4,345 proteins. The proteins in our database were only human proteins, which may lead to the condition that many compounds searched return no results as these compounds only act on non-human proteins. For these 44 compounds, 43 of whose related proteins could be searched in the TCM-Mesh with corresponding genes been retrieved. We have collected 4,954 pairs of associations between 43 compounds and 3,713 genes finally.

**Figure 4:**
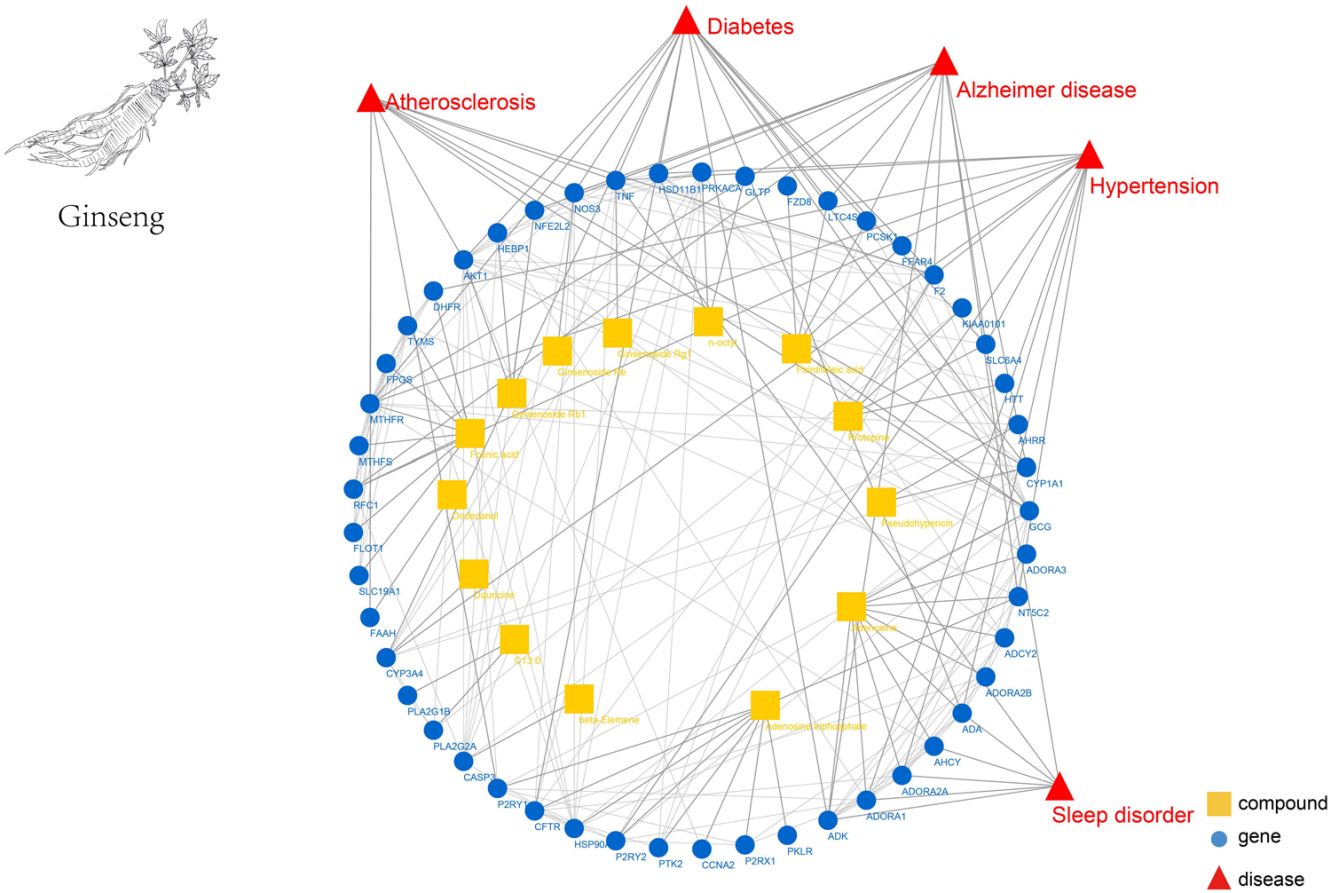
The pharmacology network generated for ginseng. The innermost circle represents the compounds collected from the ginseng, the circle in the middle represented the related genes of compounds from ginseng, the outermost layer represented the diseases that were related to the genes. Node: yellow square, compound; blue circle, gene; red triangle, disease. Line: the line between yellow square and blue circle, compound-gene links; the line between blue circle and red triangle, potential links between gene and disease. The ginseng in the figure was drawn in-house according to the real ginseng.

We set the threshold of combined score to 700, and we obtained 2,547 pairs of associations between 14 compounds and 2,221 genes. These 14 compounds were Adenosine, Adenosine triphosphate, beta-Elemene, Dauricine, Dodecanol, Folinic acid, Ginsenoside Rb1, Ginsenoside Re, Ginsenoside Rg1, n-octyl, Palmitoleic acid, Protopine, Pseudohypericin and Tridecanoic acid respectively. We found that 3 out of 14 compounds were ginsenosides, which were regarded as the main active components of ginseng. We chose 10 genes with the highest score for each compound and 49 genes were retained. In addition, we also collected 121 pairs of gene interactions. And we have found that by targeting some of these 49 genes, ginseng could exert effects on several diseases including Alzheimer disease, atherosclerosis, hypertension, sleep-disorder and diabetes. From the network (Fig. 4), three ginsenosides were related to the most indication of the ginseng, which also demonstrated the pharmacological activity of ginsenoside as we mentioned above. To further validate the “TCM-compounds-proteins/genes-diseases” network generated for ginseng, we investigated whether the main active components of ginseng, i.e. ginsenosides, has been reported to be related to the diseases arise from the network. As a result, it has been reported that the Ginsenoside Rg1 related gene, i.e. TNF was related to diabetes, Alzheimer’s disease, hypertension and atherosclerosis^57–61^. In addition, the Ginsenoside Re related gene, i.e. NOS3 was associated with the Alzheimer’s disease^62^. Moreover, AKT1, a Ginsenoside Rb1 related gene, was related to diabetes. Furthermore, some related genes of other compounds of ginseng collected from the TCM-Mesh such as ADA, ADK, ADORA2A and GCG were reported to be associated with the sleep disorder^63, 64^ and diabetes^65–68^.

#### Assessment of networks generated for LDW

We obtained 224 compounds of LDW from the TCM database@Taiwan and TCMID. As a result, 76 compounds could be used for searching in TCM-Mesh with 6,775 pairs of associations between 76 compounds and 3,982 proteins been retrieved. Then we used protein collected from TCM-Mesh for searching, and we obtained 6,219 pairs of associations between 76 compounds and 3,335 genes finally.

**Figure 5:**
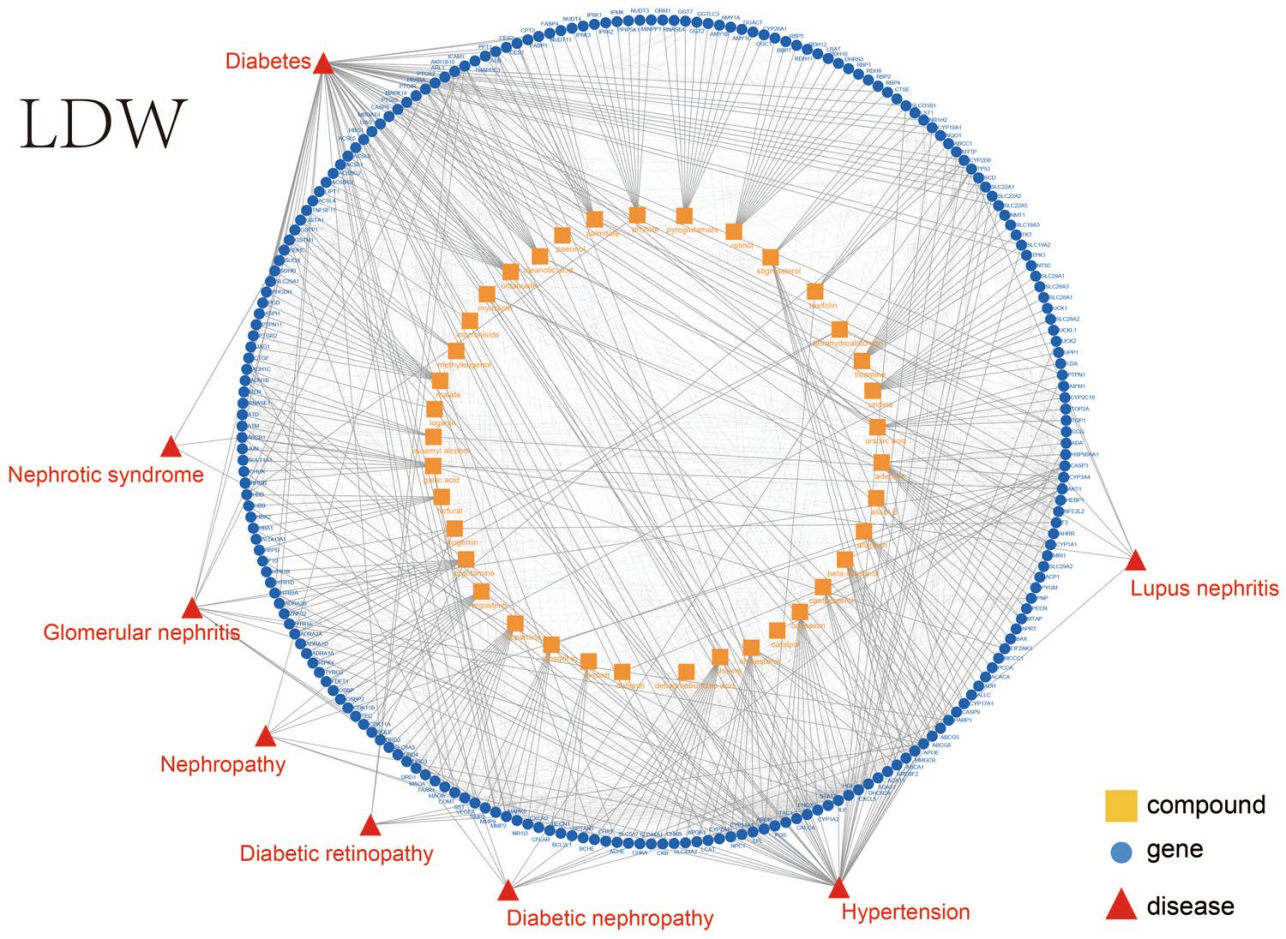
The pharmacology network generated for LDW. The innermost circle represented the compounds collected from LDW, the circle in the middle represented the related genes of compounds from LDW, the outermost layer represented the diseases that were related to the genes. Node: yellow square, compound; blue circle, gene; red triangle, disease. Line: the line between yellow square and blue circle, compound-gene links; the line between blue circle and red triangle, potential links between gene and disease.

We set combined score to 700 with 1,313 pairs of associations between 38 compounds and 980 genes been retrieved from the TCM-Mesh. For each compound, we chose 10 genes with the highest score and as a result 206 genes were retained. Moreover, we collected 1,200 pairs of gene interactions. We found some bioactive constituents of LDW, i.e. morroniside, gallic acid, loganin and paeonol^16, 69^, were in these 38 compounds. And the content of loganin, as well as paeonol were regarded as the quality control indexes of LDW in Chinese Pharmacopoeia. From the network (Fig. 5), we found that loganin was related to diabetes and diabetic nephropathy. In addition, paeonol was associated with diabetes, diabetic nephropathy and diabetic retinopathy. To validate the candidate “TCM-compounds-genes-diseases” pathways generated for LDW, we investigated whether these quality control indexes have been reported to be associated with the diseases arise from the network. As a result, paeonol related gene, i.e. ICAM1 was related to diabetes, diabetic nephropathy and diabetic retinopathy^70–73^, which was correspond to our results. And the loganin related gene, i.e. CTGF was associated with diabetes and diabetic nephropathy^74–78^, which was also consistent with our results.

In summary, the compounds of the TCM and TCM preparation that played a chief part in therapeutic effects have been validated by literature. These results may offer a pathway for the molecule of Ginseng and LDW to exert their effects.

#### Network comparison

In addition to the self-evaluation of the network, we also compared the network of TCM-Mesh with TCMID, with ginseng as an example. According to the TCMID, the network it generated for ginseng contained 4,820 pairs of associations between ingredients and targets, and it contained 2,813 pairs of associations between targets and diseases. For TCM-Mesh, to completely display the network of ginseng, we set combined score to 0. As a result, the network TCM-Mesh generated for ginseng contained 4,144 pairs of associations between ingredients and targets, and it contained 2,969 pairs of associations between targets and diseases. In contrast to TCMID, we also offered interactions between targets, which was not shown in TCMID, it contained 183,777 pairs of targets interactions. Compared to the network of TCMID which contained both the compound with and without targets, the network of TCM-Mesh only retained the compounds with targets, thus users could focus on TCM’s “compound-proteins/genes-diseases” pathways.

### Searching efficiency of the database

100 FDA approved drugs were selected randomly from the 1,293 FDA-approved drugs mentioned above to test the efficiency of the database. As the database could save the result of the first searching as cache, the second query consume less time. We set the threshold of combined score to 600 manually for simplicity. For all the 100 drugs, the average searching time was 0.5844 s and 0.5067 s for the first and second query respectively. The searching speed increased by 13.3% at the second searching against the first time on average.

To further study the efficiency of the database and figure out which step of the whole process spent more time, we selected 100 FDA approved drugs randomly from the FDA-approved drugs mentioned above and recorded the time of each step consuming in our internal database. There were three major steps, i.e. searching proteins by using the name of compound, searching genes by using proteins, as well as searching diseases by using genes, which would be taken into consideration. And the average time spent in three steps were nearly the same, they were 0.0296, 0.0253 and 0.0225 respectively.

**Figure 6:**
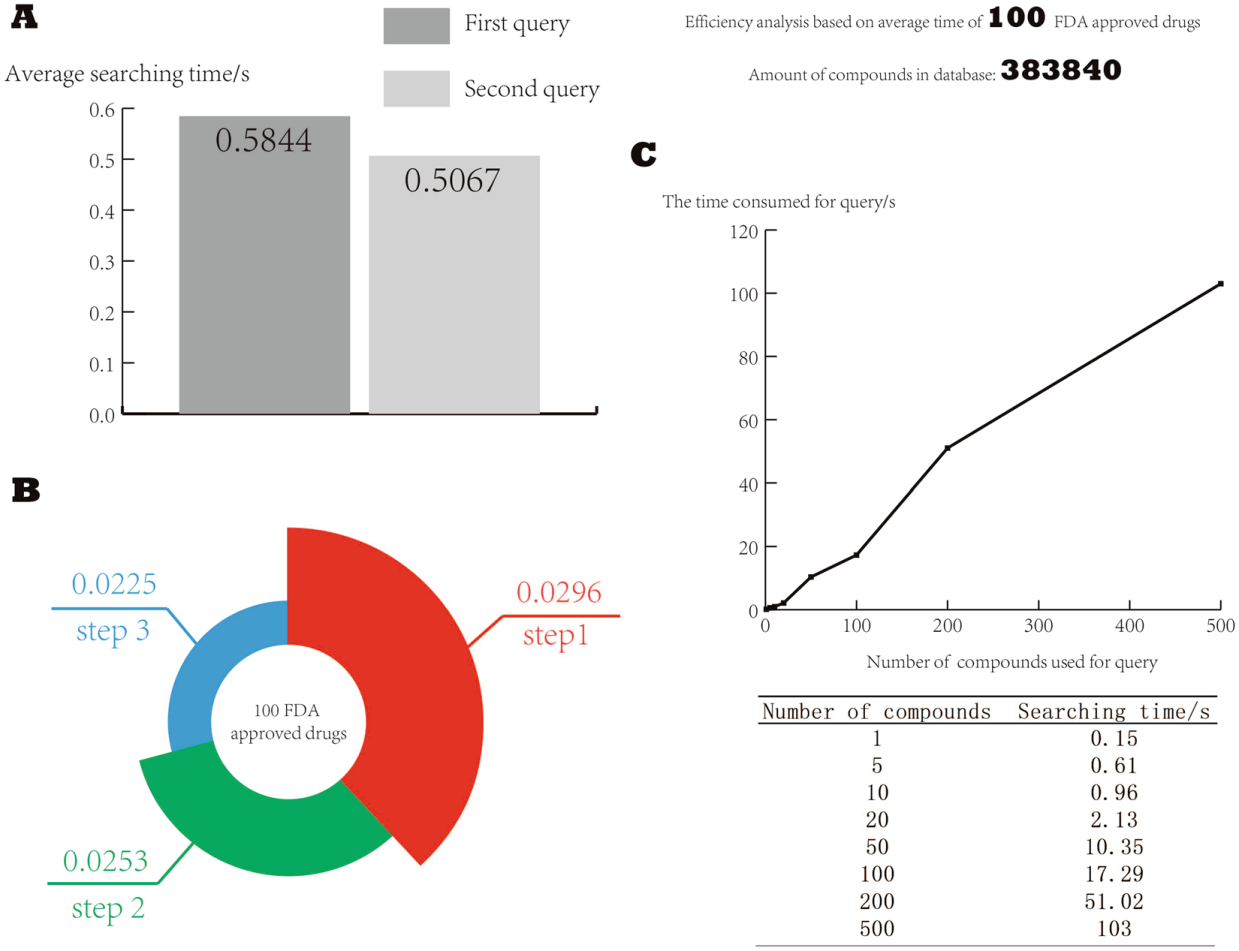
The searching efficiency of the database. (**A**) Time consumed of the first query and the second query; (**B**) Time spent on different steps of the whole process; (**C**) Searching time of searching with different amount of compounds.

Moreover, we used the different amount of compounds to search in our database, i.e. 1, 5, 10, 20, 50, 100, 200 and 500 compounds respectively. And the time-consuming were 0.15 s, 0.61 s, 0.96 s, 2.13 s, 10.35 s, 17.29 s, 51.02 s, 103 s respectively. As shown in Fig. 6, with the increasing of the amount of compounds, retrieval efficiency was not significantly affected.

## Conclusion

The contemporary approach for TCM preparation effectiveness analysis is systems biology, based on which the “small molecule-proteins/genes-diseases” chains could be established. However, current network pharmacology approaches are limited by their completeness and the simple analytical method.

We have designed the TCM-Mesh system, which contained 6,235 herbs, 383,840 compounds, 14,298 genes, 6,204 diseases, 144,723 gene-disease associations, 3,440,231 pairs of gene interactions, 163,221 side effect records and 71 toxic records. In order to evaluate the completeness and the accuracy of the database, we used eight intestinal disease-related drugs and 1,293 FDA approved drugs for testing. And we have found the potential “compound-proteins/genes-diseases” pathways for all eight intestinal disease-related drugs. In addition, 1,015 out of 1,293 FDA-approved drugs with related genes were collected from the TCM-Mesh when we set combined score to 700, and the related genes of 81.6% drugs have been found to be related to the indications of the drugs. The fact that nearly 20% of the drugs (187 out of 1,015) were searched in the TCM-Mesh and no candidate target were retrieved might be due to two reasons: Firstly, we have found that some drugs were not used for treatment of diseases, but used as auxiliary agents such as anesthetic and contrast medium, thus these kinds of drugs could not be searched in the TCM-Mesh, which was focus on the diseases; moreover, for each compound we chose 10 genes for simplicity, therefore, possible targets might be filtered.

We have compared TCM-Mesh database with several public available databases, and we have found that our database obtained the same searching results with STITCH while our database was much conciser. Furthermore, our database was relatively more complete compared to TCMID. As a database mainly focusing on TCM ingredient analysis, TCM-Mesh’s comparable results with those based on the general database such as STITCH has shown its superiority for TCM ingredient interpretations.

Based on the TCM-Mesh system, we have tested and interpreted the functions of a famous traditional herb ginseng and a widely used TCM preparation: Liuwei Dihuang Wan, from the network perspective, and we have found the candidate pathway for bioactive components of TCMs to exert the respective pharmacological effect.

The TCM-Mesh system has its web portal for searching of TCM preparations, small molecules, as well as genes and diseases. Results have shown that comprehensive information on herbs, compounds, genes, diseases, as well as relationship between them, would be provided. And the downloadable result report for TCM preparation and drugs were also offered for the users.

### Future perspectives

TCM quality control, as well as better understanding of the regulation of TCM preparations on diseases, would be essential for the application of TCMs. Firstly, we used the combined score in the current version of TCM-Mesh for simplicity, which was still primitive and did not reflect the fidelity of the “compound-proteins/genes-diseases” pathways, and refined scoring function will be added in the future. Secondly, with the advancement of weighted network analysis method, we believe that deeper mining of key chains in “small molecule-proteins/genes-diseases” network would be critical, which will be integrated into our next version. Thirdly, a holistic view about network pharmacology should be established as soon as possible: gut microbial communities act both as targets of drugs, as well as drugs themselves that could affect human as the host^79^. Only by integration of gut microbiota, could we obtain a full picture of the TCM effectiveness map of TCM preparations.

## Materials and Methods

### Data sources

TCM-Mesh was composed of four data fields (Table 4), namely herb-ingredients, compounds, proteins and diseases. In the current version, all the information and data were integrated based on related web-based databases.

**Table 4:** Data sources of the TCM-Mesh and detailed information of different databases. The data were mainly divided into four groups: Herb-ingredients links, compounds, proteins and diseases, the totally data were obtained from seven authentic databases: TCMID, STITCH, STRING, OMIM, GAD, TOXNET and SIDER. The size of each database, the description of every database and the website links of all databases were shown.

Data field	Data source	Amount of data	description	link
Herb-ingredients	TCMID	57,249	Herbs and corresponding ingredients	http://www.megabionet.org/tcmid/
Compounds	STITCH	82,841,024	Compound-CID number links	http://stitch.embl.de/
	TOXNET	103	Compound’s toxicity	https://toxnet.nlm.nih.gov/
	SIDER	163,221	Compound’s side effect	http://sideeffects.embl.de/
Proteins	STITCH	4,523,609	CID number-protein links	http://stitch.embl.de/
	STRING	4,274,001	Protein interactions	http://www.string-db.org/
	OMIM	2,449,433	Proteins and corresponding aliases	http://omim.org/
	OMIM	15,591	Approved gene and MIM number	
Diseases	OMIM	7,086	MIM number-disease links	
	GAD	167,130	Gene-disease associations	https://geneticassociationdb.nih.gov/

#### Small molecular drug

For a small molecular drug, we could set a threshold of combined score with the “compound-proteins/genes-disease” network obtained from our database. If there were more than 10 related genes in our result, we only retained the ten genes with the highest combined score for simplicity. The information of the drug and its corresponding indications were collected from the Drugbank^56^. Then we compared the indications with the disease from the network. If one of the diseases matched with recorded indications, we might find a candidate pathway for the drug to exert its effect.

#### Traditional Chinese herb

Traditional Chinese plant consisted of a great number of different small molecules. The chemical constituents of traditional Chinese medicine were obtained from the TCM Database@Taiwan^32^ and TCMID^34^.

After obtaining all the processed compound files, we used the Can SMILE string or name of small molecules to search with the related genes and corresponding potential related-disease retrieved from our database. Unlike the small molecular drug, we took all the traditional Chinese medicine related compounds as a whole. It relied on different compounds’ synergistic effects to exert its effect. Different compounds may act on the same pathway.

#### Traditional Chinese medicine preparation

Traditional Chinese medicine preparation consisted of several different traditional Chinese medicine. The different traditional Chinese medicine related molecule data were also obtained from the TCM Database@Taiwan and TCMID. The detailed data analysis process was the same as that for traditional Chinese herb.

#### Consistency consideration

The information of herb-ingredients such as herb and its related compounds with CID number were collected from TCMID. The information of compounds and their targets, diseases and their related proteins were obtained from STITCH and OMIM respectively. As the target ID used by STITCH and OMIM were different from each other, the data from these resources were inconsistent. To overcome the barriers, the information of proteins aliases and approved gene symbol were retrieved from OMIM to unite the data. The protein interactions were obtained from STRING. The toxic and side-effect records of compounds were derived from TOXNET and SIDER. And the gene-disease associations were collected from GAD for facilitating the analysis. The FDA-approved compounds used in our study were retrieved from DrugBank, while the chemical constituents of Ginseng were collected from the TCM Database@Taiwan and chemical constituents of LDW were collected from TCM Database@Taiwan and TCMID.

### Data integration and filtration, manual annotations and corrections

The compounds of ginseng and LDW that we downloaded from TCM Database@Taiwan were mol2 formats while the formats we used in STITCH were SMILES formats. To improve compatibility of data, we converted all of them into a standard generalized format: Canonical SMILE format (Can). We used Open Babel^80^ to convert both mol2 and SMILE formats into CAN formats. To further unify the format, the symbols represented configuration and chirality like “/”, “\”, “@”, “[]” and “H” were removed from the CAN format in both the database and the herb related compounds. With the unified format obtained, the Can SMILE string of compounds could be used to search with hits from the TCM-Mesh.

**Figure 7:**
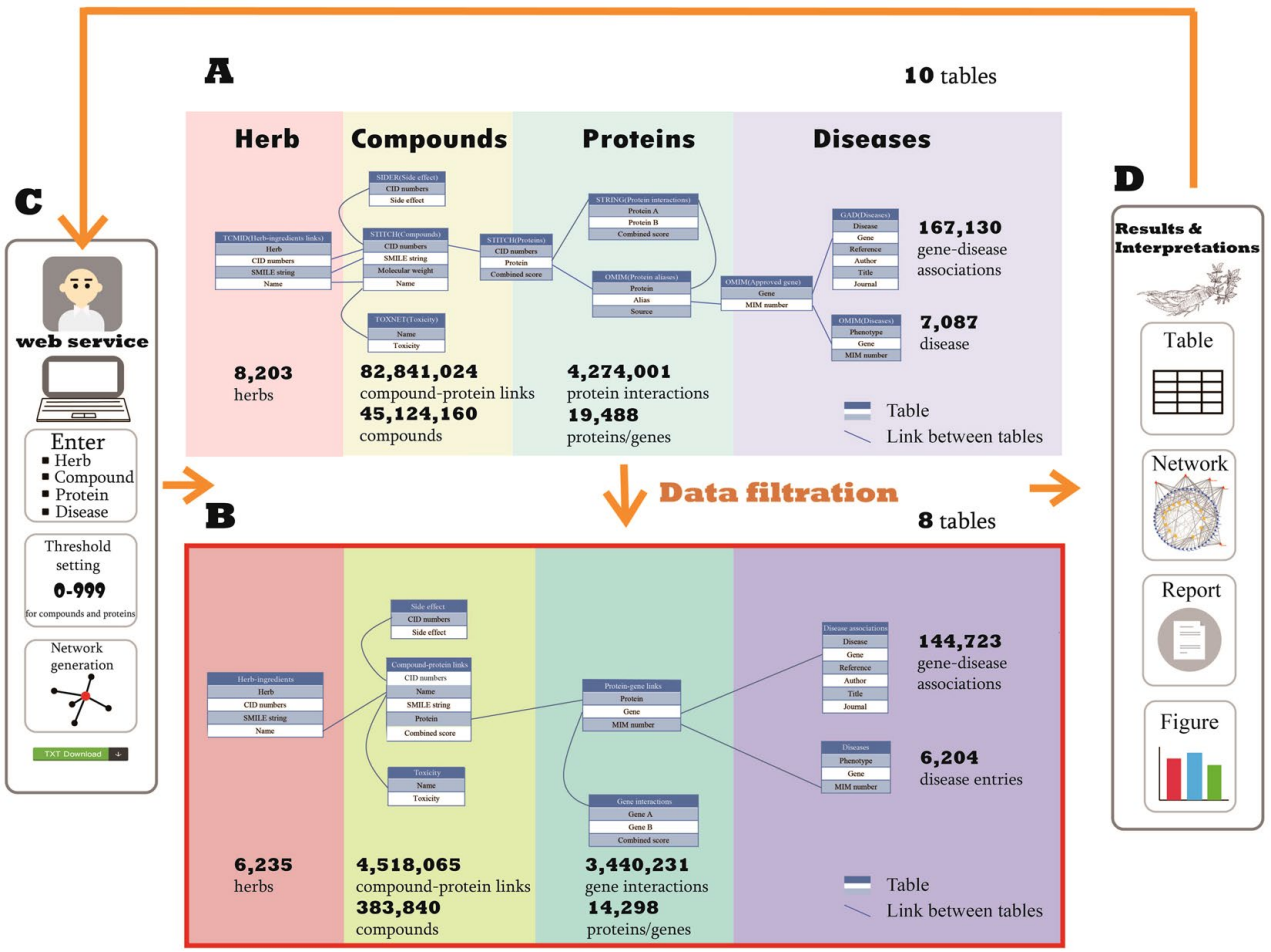
The data organization scheme and search pipeline of TCM-Mesh. (**A**) the source data of the TCM-Mesh, the raw data was consisted of ten data fields; (**B**) the filtered data of the TCM-Mesh, the filtered data consisted of eight data fields, and the size of data was reduced significantly compared with the previous database shown in (**A)**; (**C**) the web service provided for TCM-Mesh, compound, protein, gene and disease could be input for searching, threshold of combined score could be set by users to filter the results, network and download functions were available; (**D**) the results and interpretation showed by TCM-Mesh, which could be use for further analysis. The ginseng in the figure was drawn in-house according to the real ginseng.

We integrated data from various data sources (Fig. 7A), to make our data structure clearer, we further simplify our database. Firstly, the CID number was an internal connection between the compound and the protein, thus we could establish direct relations of compound and proteins. The original two tables contained up to 82,841,024 entries and 4,523,609 entries respectively, and data size of the new table named “compound-protein links” was reduced to 4,518,065 entries (Fig. 6B), with compounds in the database also reduced from 45,124,160 to 383,840 (referred to as the “filtered compound table”, Fig. 7B). In addition, the data filed about herb-ingredients contained many compounds without SMILE string and CID number, which could not be used for further searching. These compounds were deleted from our data and the number of herbs was reduced from 8,203 to 6,235.

Secondly, target ID was different in various databases as we mentioned above. To unite our data, we used the protein aliases from OMIM to establish relation between the proteins and the genes, thus for most of proteins with the corresponding genes of proteins been retrieved from our database. The original table named protein aliases and approved gene were merged into one new table called “protein-gene links”, which contained 14,298 genes. And protein interactions were also converted to the genes interactions. Thus the amount of data was reduced from 4,274,001 entries to 3,440,231 entries as many proteins was searched with no results return.

Finally, the GAD contained 167,130 entries, as many diseases were redundant in the database, we processed our data manually and removed the duplicate records, as a result the amount of data were reduced to 144,723 entries. Similarly, the disease entries of OMIM were reduced from 7,086 to 6,204.

### Constructing the TCM-Mesh database based on MySQL

The TCM-Mesh database was built by MySQL. The main goal of our database was to build the connection between the herb and diseases. And the various data fields in our database were connected with their intrinsic relations (Fig. 7B).

Firstly, we used the information of herb supplied by TCMID to connect herbs and their respective ingredients. We used the name and the CID numbers of compounds to search with the toxicity and the side effect of the compounds, as well as the related proteins of the compounds been retrieved from the TCM-Mesh. Secondly, we used the proteins to search and corresponding gene, gene interactions were retrieved. Finally, we used the genes to search in the database and obtained the candidate related-disease and the gene-disease associations. We have used combined score between compound and protein as a threshold to filter the results.

### Generation of pharmacological network

The “compound-gene-disease” network was visualized by using Cytoscape^81^, an open source platform for complex network analysis and visualization, which would help to display the network in a more interactive manner. The compound in the network was shown as yellow square, the gene in the network was shown as blue circle while the disease was shown as the red triangle. And the association between genes was indicated by a solid line with 50% transparency while the links between compounds, genes and diseases were indicated by a gray solid line. The “yellow square-blue circle-red triangle” chain was the potential pathway for the compound to act on the disease by targeting the gene.

### Web portal

**Figure 8:**
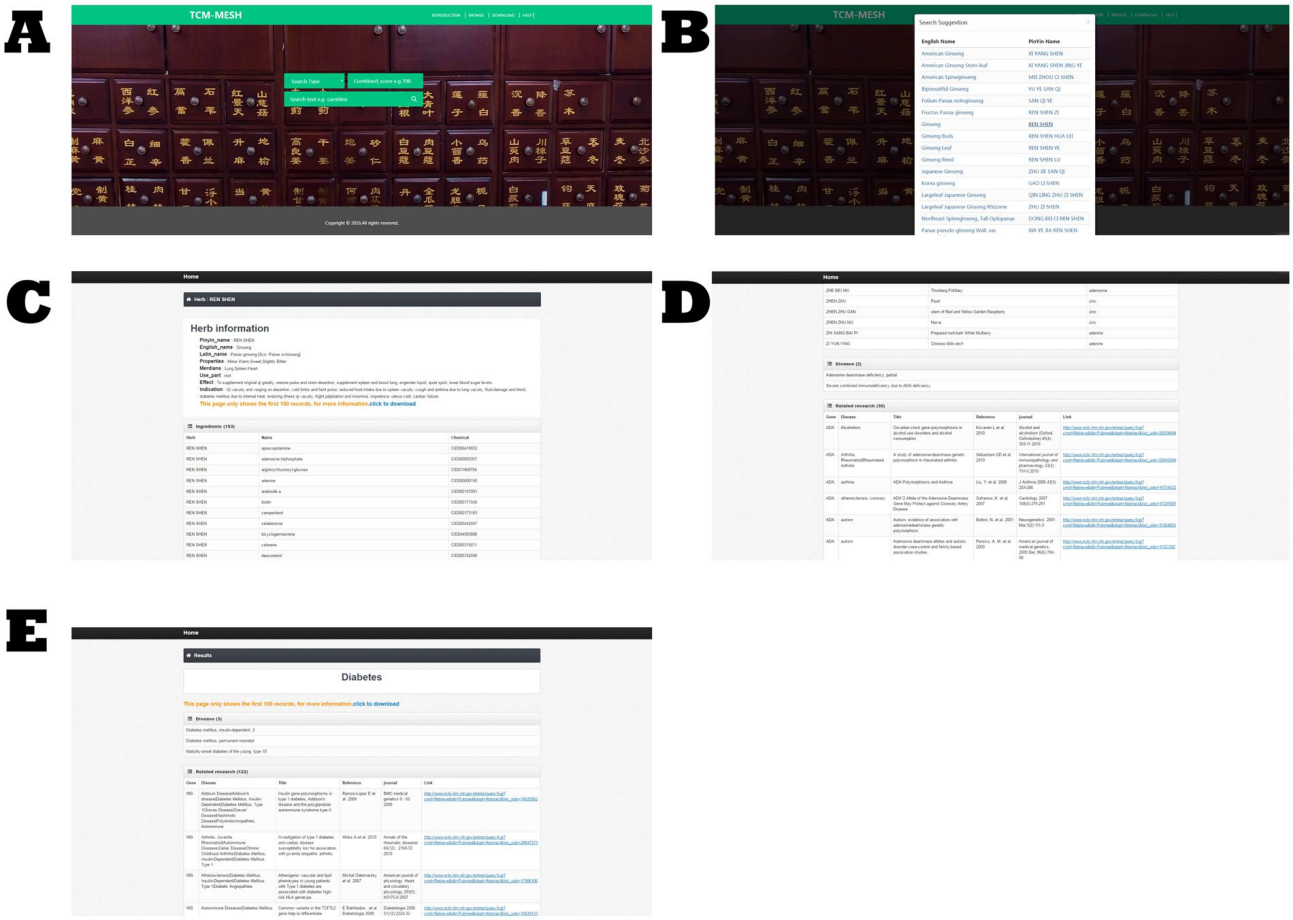
The home page and the result page of the web service. (**A**) the home page of the web of TCM-Mesh, the users could input herb, compound, gene and disease to search with the corresponding results been retrieved. (**B**) the searching suggestion when searched for ginseng. (**C**) the result page of the web when searched “ren shen”(ginseng) and the combined score was set to 700, the page showed the basic information of ginseng, the herb ingredients, the toxicity and side effect of compounds that ginseng contained and the ginseng gene-related diseases. (**D**) The ginseng related gene ADA was searched in the web and its related herbs, compounds, diseases and gene-disease associations were listed on the page. (**E**) The disease “Diabetes” and the gene “INS” were searched in the web, the INS related disease and the gene-disease associations were shown.

The web portal of TCM-Mesh was also established (http://mesh.tcm.microbioinformatics.org/). Currently, TCM-Mesh web portal has several major functions: (1) User can obtain the “herb-compounds-genes-diseases” network from the TCM-Mesh by inputting the English/pin yin name of herb. (2) Similarly, user can obtain the “compound-genes-diseases” network by inputting the name/CAN string of compound. (3) User can obtain gene related herbs, gene related compounds and gene related diseases by inputting the gene. (4) User can obtain disease related gene and disease-related researches by inputting the disease. (5) Searching suggestion is available when inputting the English/pin yin name of the herb. (6) In addition to the search function, browse, as well as download functions were made available for user. We have used “ren shen”(ginseng) as an example for testing our web service, the results webpages were shown in Fig. 8.
